# Characterizing the Copy Number Variation of Non-Coding RNAs Reveals Potential Therapeutic Targets and Prognostic Markers of LUSC

**DOI:** 10.3389/fgene.2021.779155

**Published:** 2021-12-01

**Authors:** Jinfeng Ning, Fengjiao Wang, Kaibin Zhu, Binxi Li, Qing Shu, Wei Liu

**Affiliations:** ^1^ Department of Thoracic Surgery, Harbin Medical University Cancer Hospital, Harbin, China; ^2^ Department of Management Science and Engineering, Harbin Engineering University, Harbin, China; ^3^ Department of Medical Imaging, Harbin Medical University Cancer Hospital, Harbin, China; ^4^ The Fourth Department of Medical Oncology, Harbin Medical University Cancer Hospital, Harbin, China

**Keywords:** copy number variation, non-coding RNAs, biomarkers, clinical prognosis, precision medicine

## Abstract

Lung squamous cell carcinoma (LUSC) has a poor clinical prognosis and a lack of available targeted therapies. Therefore, there is an urgent need to identify novel prognostic markers and therapeutic targets to assist in the diagnosis and treatment of LUSC. With the development of high-throughput sequencing technology, integrated analysis of multi-omics data will provide annotation of pathogenic non-coding variants and the role of non-coding sequence variants in cancers. Here, we integrated RNA-seq profiles and copy number variation (CNV) data to study the effects of non-coding variations on gene regulatory network. Furthermore, the 372 long non-coding RNAs (lncRNA) regulated by CNV were used as candidate genes, which could be used as biomarkers for clinical application. Nine lncRNAs including *LINC00896*, *MCM8-AS1*, *LINC01251*, *LNX1-AS1*, *GPRC5D-AS1*, *CTD-2350J17.1*, *LINC01133*, *LINC01121*, and *AC073130.1* were recognized as prognostic markers for LUSC. By exploring the association of the prognosis-related lncRNAs (pr-lncRNAs) with immune cell infiltration, *GPRC5D-AS1* and *LINC01133* were highlighted as markers of the immunosuppressive microenvironment. Additionally, the cascade response of pr-lncRNA-CNV-mRNA-physiological functions was revealed. Taken together, the identification of prognostic markers and carcinogenic regulatory mechanisms will contribute to the individualized treatment for LUSC and promote the development of precision medicine.

## Background

Lung cancer is the most common cause of cancer-related deaths worldwide (Ferlay et al., 2015), although our understanding of the pathogenesis and treatment options of lung cancer has improved. Lung squamous cell carcinoma (LUSC) is a subtype of non-small cell carcinoma, which accounts for about 40% of lung cancers (Goldstraw et al., 2011). Compared with lung adenocarcinoma (LUAD), another subtype of non-small cell lung cancer (NSCLC), LUSC has a poor prognosis and lacks effective clinical drugs (Hirsch et al., 2017). Immune checkpoint blockade (ICB) therapy is a hot spot in cancer treatment (Yu et al., 2016). However, this treatment only works in a subset of patients for LUSC and improves the prognosis of patients (Yuan et al., 2021). Currently, histopathological images are the main method for clinical diagnosis of lung cancer (Cui et al., 2020); it is thus necessary to identify novel prognostic markers and therapeutic targets to assist the clinical treatment of LUSC.

Long non-coding RNAs (lncRNAs), which is defined as an ncRNA of at least 200 nucleotides (nt) in length (Wilusz et al., 2009; Ulitsky, 2016), have been found to play a critical role in the regulation of gene expression contributing to physiological function homeostasis, aging, and multiple cancers (Rinn and Chang, 2012; Schmitt and Chang, 2016). With the development of high-throughput technologies and comprehensive databases (Wang et al., 2019a; Wang et al., 2021), the integrated analysis of multiple omics data to reveal the roles of lncRNAs in tumor pathogenesis has become the norm. Genetic variation in the lncRNA region, such as single-nucleotide variation, somatic mutation, and copy number variation (CNV), may affect the expression level of the gene and its target genes, which may contribute to tumor occurrence and development (Wang et al., 2020). For example, lncRNAs with CNVs drive transcriptional perturbed functional pathways (Xu et al., 2020), and single-nucleotide variation in lncRNA regulates cancer-related pathways through ceRNA mechanism (Zhang et al., 2021).

Immune cells are an important part of the tumor microenvironment (TME) and have been proven to play an important role in tumor proliferation and metastasis. For example, increased abundance of specific T cell subtypes in cancer tissues is associated with better patient prognosis (Jiang et al., 2019; van der Leun et al., 2020). Macrophage polarization plays a critical role in subverting adaptive immunity and promoting tumor progression (Mantovani et al., 2002). Exploring the relationship between dysregulated lncRNA and immune cells is an important direction for deciphering the carcinogenic mechanism of lncRNA.

Here, we collected RNA-seq profiles and CNV data of 482 tumor samples for LUSC from The Cancer Genome Atlas (TCGA) (Tomczak et al., 2015) database to identify lncRNAs whose expression is driven by CNV. Then, Cox and least absolute shrinkage and selection operator (LASSO) regression analyses were performed. Prognosis-related lncRNAs (pr-lncRNAs) driven by CNV were recognized as prognostic markers of LUSC. Moreover, we comprehensively characterized the function and regulatory mechanism of pr-lncRNA by immuno-infiltration and functional enrichment analysis.

## Methods

### Data Collection and Pre-Processing

The RNA-seq profiles (482 tumor samples), CNV data (570 tumor samples), and clinical information of LUSC collected by TCGA database were downloaded from UCSC Xena browser (https://xenabrowser.net/). The genome-wide annotation data of GRCH38 V37 including location of lncRNA was collected from the GENCODE (Frankish et al., 2021) database (https://www.gencodegenes.org/). The independent non-small cell lung cancer datasets including GSE37745 and GSE50081 were obtained from publicly available Gene Expression Omnibus (GEO, available at https://www.ncbi.nlm.nih.gov/geo/) (Okayama et al., 2012), which were used for verification of prognostic markers. The data of lncRNA-immune cell infiltration correlation was collected from the ImmLnc (Li et al., 2020) database (http://bio-bigdata.hrbmu.edu.cn/ImmLnc/). Additionally, the signature matrix of 22 types of immune cell was collected from previous studies (Newman et al., 2015) for the analysis of immune cell infiltration. For CNV data of LUSC, the software of GISTIC was used to analyze the CNV of the entire lncRNA genome, taking the number of copies greater than 1 as the threshold of copy amplification and less than −1 as the threshold of copy loss. The R package maftools (v2.4.12) (Mayakonda et al., 2018) was used to observe the distribution of the *G*-score of copy amplification and loss in the genome.

### Identification of LncRNA Driven by CNV

Based on the copy number profiles for lncRNA, lncRNAs that have no overlapped segments in more than 50% of patients were deleted. We grouped patients according to the degree of copy number variation in a particular lncRNA region, and patients with copy numbers greater than 1 or less than −1 were assigned to the group of patients with CNVs, while other patients were assigned to the control group. Then, the Mann–Whitney *U* test was used to test whether the lncRNA expression was differentially expressed in the group of patients with CNVs (H0: there was no difference between the group of patients with CNVs and the control group). We also calculated the fold change (FC) of each lncRNA between the group of patients with CNVs and the control group, i.e., $FC=\;m_{cnv}/m_{c}$ , where $m_{cnv}$ and $m_{c}$ represent the mean expression of an lncRNA in the group of patients with CNVs and the control group, respectively. The lncRNAs with *p* < 0.01 and |FC| > 0 were considered to be lncRNAs driven by CNV.

### Identification of Prognosis-Related LncRNA Signature

Based on the lncRNAs driven by CNV, we generated the lncRNA expression matrix. The univariate Cox regression using R package survival (Guo et al., 2019) was employed to screen for lncRNAs with expression level significantly associated with patient’s overall survival (OS) of LUSC patients (the cutoff of *p*-value was 0.05). Furthermore, the LASSO regression using R package glmnet (v4.0-2) (Alhamzawi and Ali, 2018) was used to further screen for pr-lncRNA driven by CNV (pr-lncRNA-CNV) based on a more rigorous algorithm. Next, we randomly selected 70% of all tumor samples as the training set and the remaining as the test set. The features selected by the LASSO regression were used to fit for a multivariate Cox risk regression model based on training sets. The reliability of the Cox risk regression model was validated by the receiver operating characteristic (ROC) curve, and the area under curve (AUC) also was calculated. Finally, we defined the lncRNAs with a *p*-value <0.05 as the pr-lncRNA signatures, which significantly contributed to the LUSC patient survival outcomes. Besides, the nomogram method was used to build a more intuitive prediction model, and the calibration curve was used to evaluate the predictive ability of nomograph on the patient’s 1- and 3-year survival risk.

### Construction of Risk Scoring Model

We used the linear combination of expression values weighted by the coefficient from the multivariate Cox regression analysis to calculate the risk score for each patient:

$$Riskscore\left(i\right)=\overset{n}{\underset{k=1}{\sum }}\beta_{k}e_{\mathrm{ki}},$$

where *n* denotes the number of the pr-lncRNAs (*n* = 9), β was the coefficient of multivariate Cox regression analysis, and $e_{\mathrm{ki}}$ was the expression level of the *k*th pr-lncRNA expression of patient *i*. The median risk score was used as the cut-off to divide patients into high- and low-risk groups. Then, the samples of training set and test set were respectively divided into high-risk and low-risk categories. Additionally, Kaplan–Meier survival curve (Ranstam and Cook, 2017) was used to prove the difference of OS between the high-risk group and low-risk categories, and the bilateral logarithmic rank test (Guyot et al., 2012) was used to calculate the statistical significance. The whole pipeline above was also performed in the independent dataset [GSE37745 (Botling et al., 2013) and GSE50081 (Der et al., 2014), Supplementary Table S1] to confirm the robustness and stability of pr-lncRNAs.

### Tumor Immune Microenvironment Analysis

First, the online tool CIBERSORTx (https://cibersortx.stanford.edu/), which is a method to characterize the cell composition of complex tissues from the gene expression profile based on signature matrix, was used to calculate the fraction of immune cell infiltration abundance. Then, the parameter perm, which is the number of permutations when calculating the *p*-value, was set to the max 1,000, and quantile normalization was disabled. The function ggboxplot from the ggpubr package visualized the difference in the abundance of 22 types of leukocytes between the high- and low-risk groups. Additionally, we identified immune cells associated with patient prognosis by linking their abundance to patient’s OS. Besides, the lncRNA–immune cell type correlation was performed by the ImmLnc.

### Construction of CNV-LncRNA-PCG Network

We first generated the expression matrix of protein-coding genes (PCGs) to calculate PCGs related to the expression of pr-lncRNA driven by CNV using Pearson’s algorithm (Bishara and Hittner, 2012). Then, we have defined that gene pairs with a *p*-value <0.01 and *R* > 0.3 correlation coefficient have significant correlation in expression and are co-expressed genes with each other. For PCGs related to each pr-lncRNA, the Mann–Whitney *U* test was used to test whether the PCG expression was differentially expressed between the group of patients with CNVs and the control group. Finally, PCGs whose expressions were both differently expressed between the two groups and correlated with pr-lncRNA’s expression were collected as signature PCGs. The CNV-lncRNA-PCG network was visualized using Cytoscape (v3.7.0) (Shannon et al., 2003).

### Functional Enrichment Analysis

PCGs driven by the CNV of each pr-lncRNA were used to annotate the biological functions of pr-lncRNAs. The functional enrichment analysis was performed by the online tool Metascape (Zhou et al., 2019) (http://metascape.org/).

### Statistical Analysis

All analyses were conducted using R (v3.6.3) software. The gene sets enrichment analysis was performed using the Fisher’s exact test. Log-rank test was used to compare the difference of survival time between two groups.

## Results

### Copy Number Amplitude Perturbs the Expression of LncRNA

**FIGURE 1:**
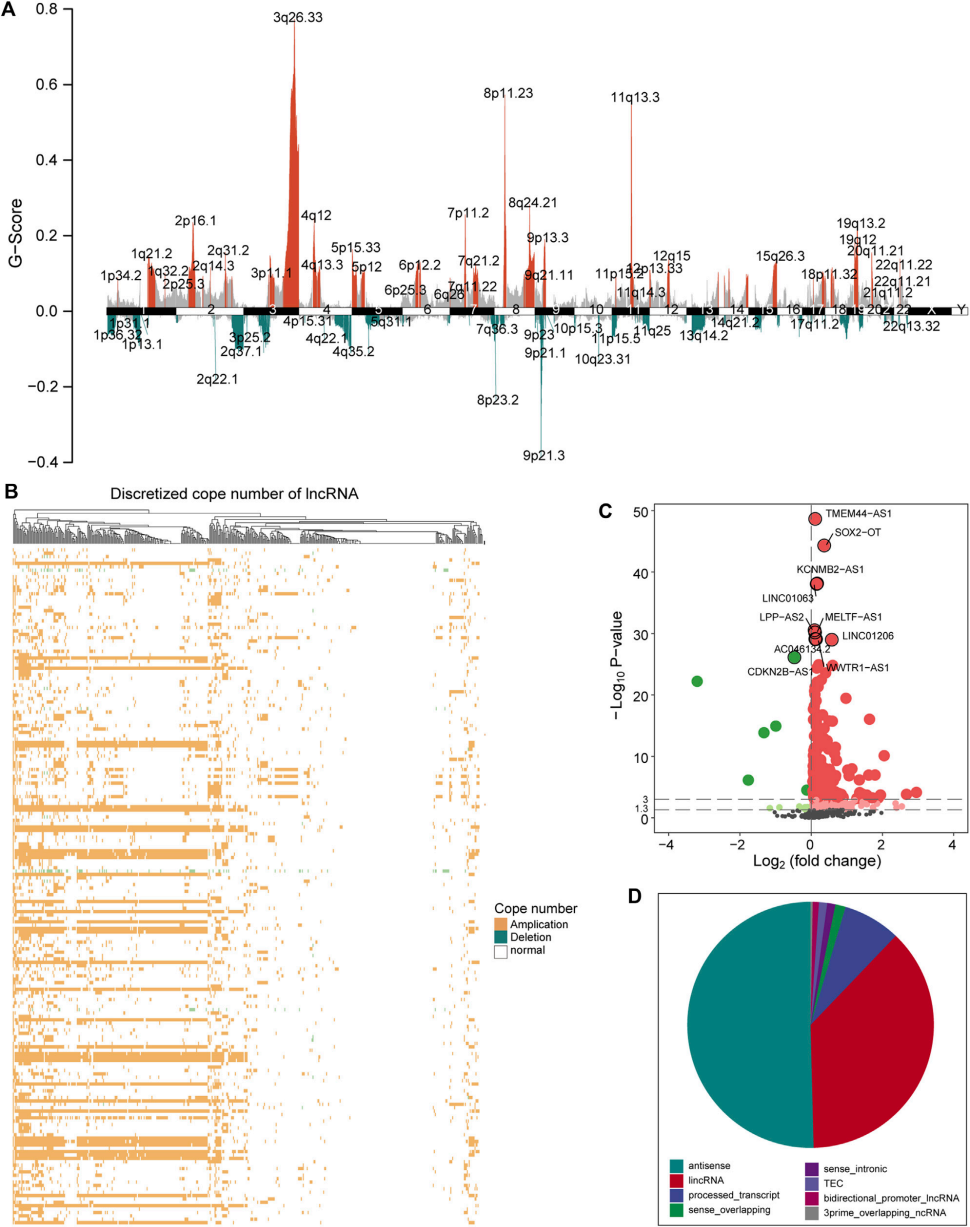
A global view of CNV for LUSC. **(A)** The graph shows the distribution of the copy number amplitude on the genome for LUSC. The ordinate represents *G*-score, and the abscissa is the position of 22 homologous chromosomes and two sex chromosomes. **(B)** The amplification and deletion of lncRNA copy number in tumor samples are displayed by a heat map. Yellow indicates copy number amplification, blue indicates copy number deletion, and white indicates no CNV has occurred. **(C)** The expression difference of the candidate lncRNA between the group with CNV in the lncRNA and the control group is shown by a volcano graph. Red dots indicate up-regulation, and green dots indicate down-regulation. **(D)** The pie chart shows the proportion of various types of CNV-driven lncRNAs. Different colors represent specific lncRNA types. CNV, copy number variation; LUSC, lung squamous cell carcinoma; lncRNA, long non-coding RNA.

Aneuploidy such as CNV is a hallmark of most solid tumors (including LUSC) (Ried et al., 2019). To explore the role of CNV on lncRNA in the carcinogenic mechanism of LUSC, we first analyzed the global characteristics of CNV. Maftool was used to visualize the copy number amplitude calculated by GISTIC on the whole genome. We found that there is an obvious copy number amplification on q26.33 of chromosome 3, p11.23/q24.21 of chromosome 8, and q.13.3 of chromosome 11 and copy number deletion on q37.1 of chromosome 2, p25.2 of chromosome 3, p23.2 of chromosome 8, and p21 of chromosome 9 (Figure 1A). Next, the copy number amplitude data was mapped to lncRNA to build copy number profiles, and lncRNAs with significant copy number amplification and deletion are defined as candidate lncRNAs (647 lncRNAs) that are used to link lncRNA expression profiles. We found the characteristics of the copy number amplitude of lncRNA in LUSC, that is, each lncRNA with CNV has global amplification or global deletion in patients (Figure 1B). Therefore, tumor patients were divided into a group with CNV in the lncRNA and a control group based on lncRNA copy number profiles. Moreover, lncRNAs tended to undergo overall copy number amplification in LUSC (Figure 1B), which is contrary to a previous study suggesting that copy number deletion pattern of the lncRNAs was widely observed in various cancer types (Xu et al., 2020). Furthermore, we identified 372 lncRNAs whose expression levels (FPKM) are driven by CNV (Figure 1C). Similar with the copy number amplitude results, most lncRNA expressions were up-regulated in the group with CNV in the lncRNA. Among them, the significantly up-regulated lncRNA SOX2-OT driven by CNV has been shown to regulate the proliferation and metastasis of multiple cancers through the ceRNA mechanism (Zhang and Li, 2019; Herrera-Solorio et al., 2021). By annotating the types of lncRNAs driven by CNV, we found that they were mainly long intergenic non-coding RNA (lincRNA) and antisense classes (Figure 1D). Taken together, the CNV feature and CNV-driven lncRNAs for LUSC were recognized.

### The Nine Pr-LncRNAs for LUSC

**FIGURE 2:**
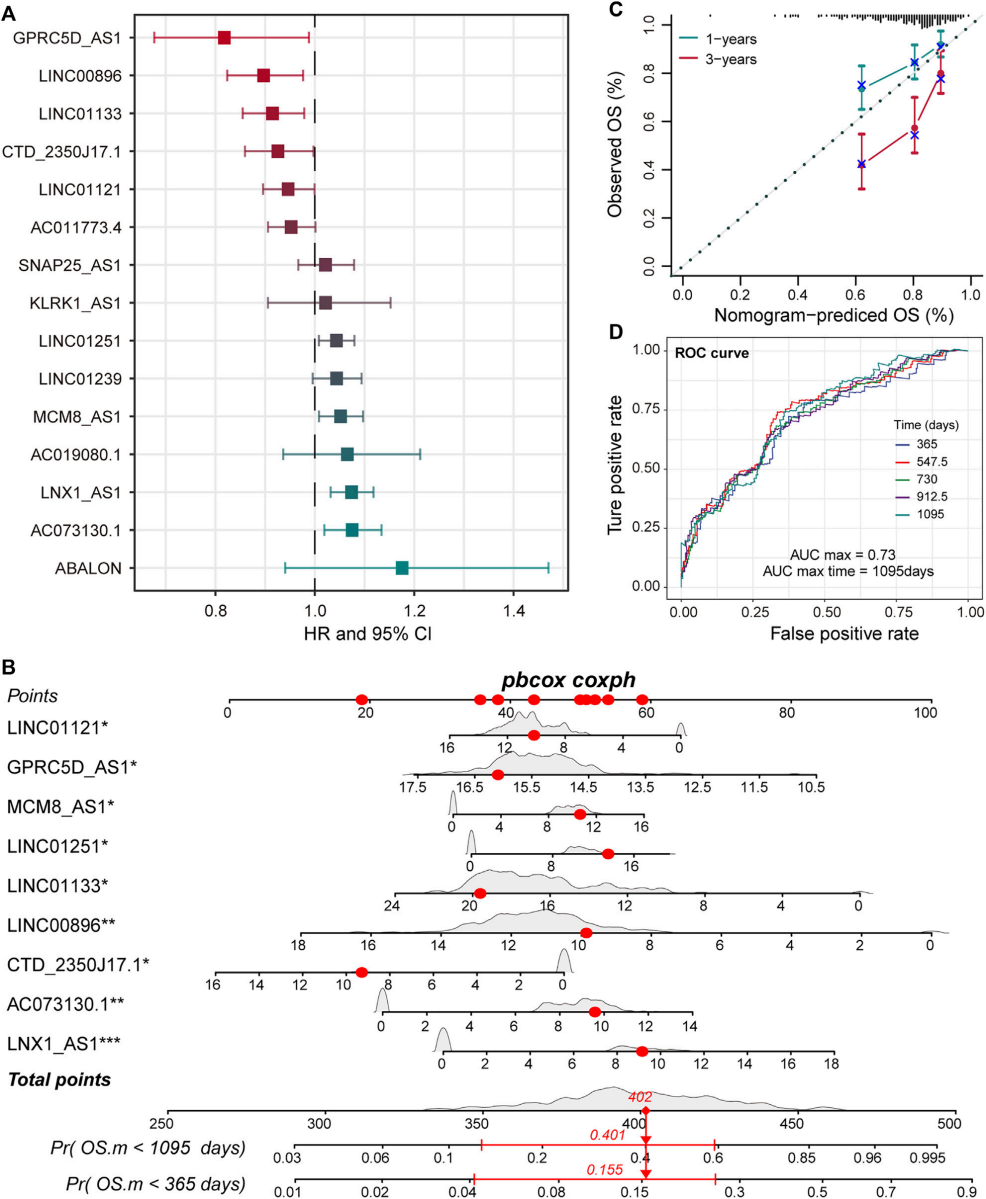
Identification of prognostic markers for LUSC. **(A)** The error bar graphs are used to show the correlation between the expression level of lncRNA and the patient’s overall survival (OS). The abscissa represents the hazard ratio (HR) value with the 95% confidence interval. **(B)** Nomogram for survival risk prediction of 1 and 3 years. The nine prognostic markers are marked by a star. Additionally, *p < 0.05, **p < 0.01, ***p < 0.001. **(C)** The calibration curve of the nomogram. **(D)** The predictive performance of the survival predictive model at five time points from 1 to 3 years. The different colored curves represent specific time points.

LncRNA is an emerging biomarker for cancer development and patient’s prognosis (Bolha et al., 2017). To identify CNV-driven lncRNAs related to prognosis, we developed a computational model by combining LASSO regression and Cox regression models to identify pr-lncRNA. We performed the univariate Cox and LASSO algorithm to identify 16 lncRNAs that were significantly related to the patient’s OS. After the construction of a multivariate Cox regression model, nine lncRNAs including *LINC00896*, *MCM8-AS1*, *LINC01251*, *LNX1-AS1*, *GPRC5D-AS1*, *CTD-2350J17.1*, *LINC01133*, *LINC01121*, and *AC073130.1* were finally identified as prognostic markers (Figure 2A, Supplementary Figure S1). For the nine pr-lncRNAs, *LNX1-AS1*, *AC073130.1*, *MCM8-AS1*, and *LINC01251* were treated as the risk factors for patient’s survival of LUSC, while *LINC00896*, *GPRC5D-AS1*, *CTD-2350J17.1*, *LINC01133*, and *LINC01121* were treated as the protective factors. Furthermore, based on the expression of nine prognostic markers, the nomogram algorithm was used to construct 1- and 3-year survival probability prediction models (Figure 2B). Then, the calibration curve was used to evaluate the predictive performance of the nomogram, and the result showed that the nomogram algorithm has a good performance in predicting the survival risk for the patients of LUSC (Figure 2C). We divide the 1–3-year period into five time points, and the ROC curve was used to determine the best prediction time point for the risk prediction model. We found that the risk prediction result reached the maximum AUC value of 0.73 in 1,095 days (Figure 2D). All these suggest that the nine pr-lncRNAs can be used as prognostic markers to predict the survival risk for patients of LUSC.

### Risk Score Model Supports the Diagnosis of Patient Prognosis

**FIGURE 3:**
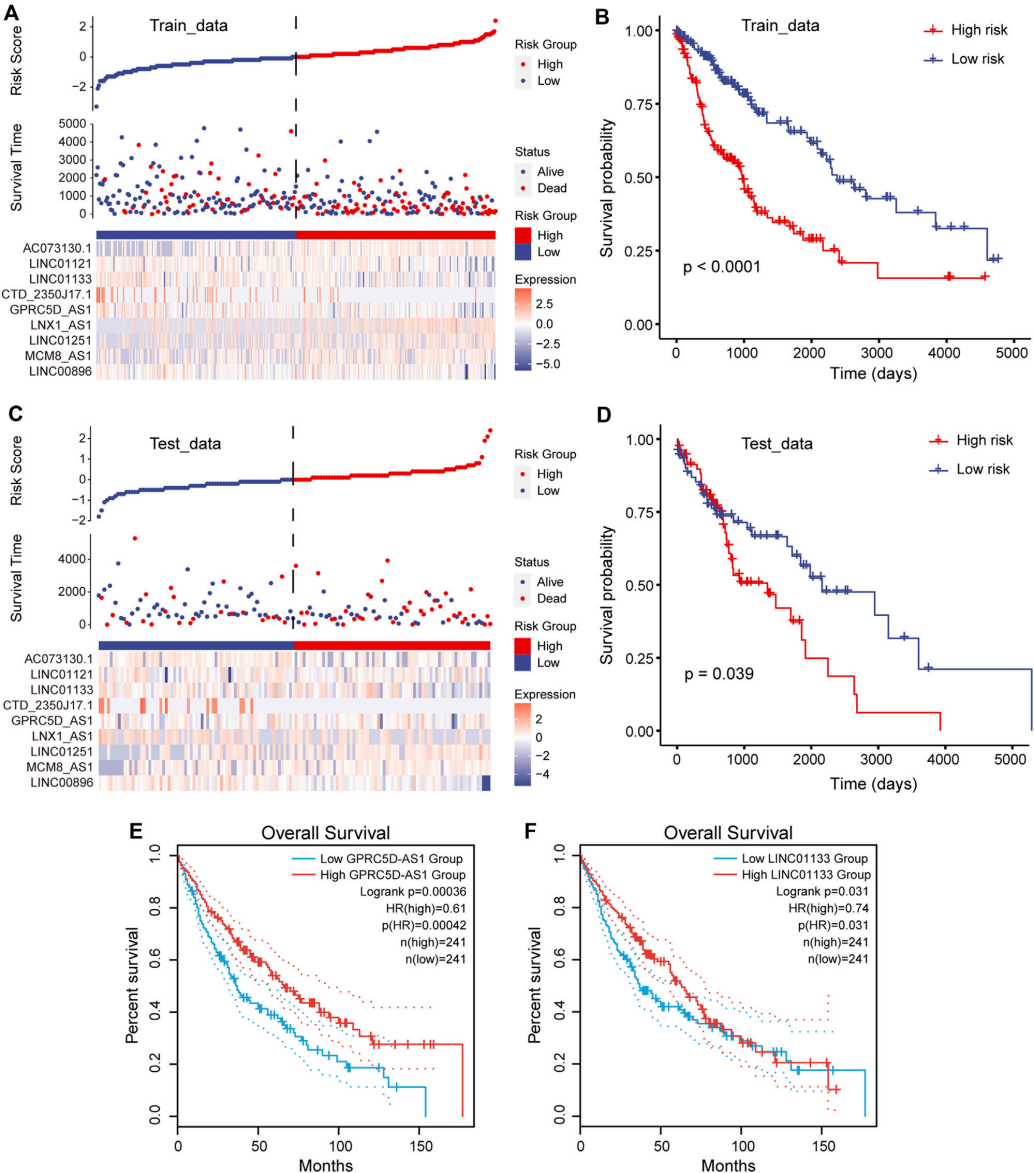
Construction of risk scoring model. **(A)** Risk scores, survival status, and the expression of nine prognosis-related lncRNAs (pr-lncRNAs) for the training set. **(B)** The Kaplan–Meier curves for the two groups in the training set. Log-rank test is used to calculate statistical significance. The unit of time is days. **(C)** Same as in **(A)** but for the testing set. **(D)** Same as in **(B)** but for the two groups in the testing set. **(E–F)** The Kaplan–Meier curves for survival in groups with high and low expression (FPKM) of lncRNA GPRC5D-AS1 and LINC01133.

To accurately quantify the survival risk of patients, we constructed the risk score model based on the regression coefficients calculated by multivariate Cox regression model and patient’s survival (Guo et al., 2020). The formula of the risk scoring model was as follows: Risk score = (−0.11 × *LINC00896* + 0.05 × *MCM8-AS1* + 0.04 × *LINC01251* + 0.07 × *LNX1-AS1* − 0.20 × *GPRC5D-AS1* − 0.08 × *CTD-2350J17.1* − 0.09 × *LINC01133* − 0.06 × *LINC01121* + 0.07 × *AC073130.1*). Furthermore, the samples of training and test sets were respectively divided into the high- and low-risk groups based on the median risk score. We found that there was an obvious expression difference of pr-lncRNAs between the high- and low-risk categories (Figure 3A), and the samples with high-risk score have poor prognosis (Figure 3B). Moreover, the high-risk samples in test sets also exhibited an association with poorer OS of LUSC (Figure 3C,D). All these suggest that the risk score can quantify the prognosis of patients and provide convenience for clinical diagnosis. Using the online analysis tool of GEPIA (Tang et al., 2019), we found that the high expression of single genes *GPRC5D-AS1* and *LINC01133* is associated with better patient prognosis (Figure 3E,F), which was consistent with the result in this study, suggesting *GPRC5D-AS1* and *LINC01133* as protective factors for patient’s OS. Besides, *LNX1_AS1* has been reported as the poor prognosis marker for non-small cell lung cancer (Wang et al., 2019b) in previous studies. The evidence further emphasized that pr-lncRNAs may play an important role in the progression of LUSC.

### Evaluation of the Robustness for Nine Pr-LncRNA Signatures

**FIGURE 4:**
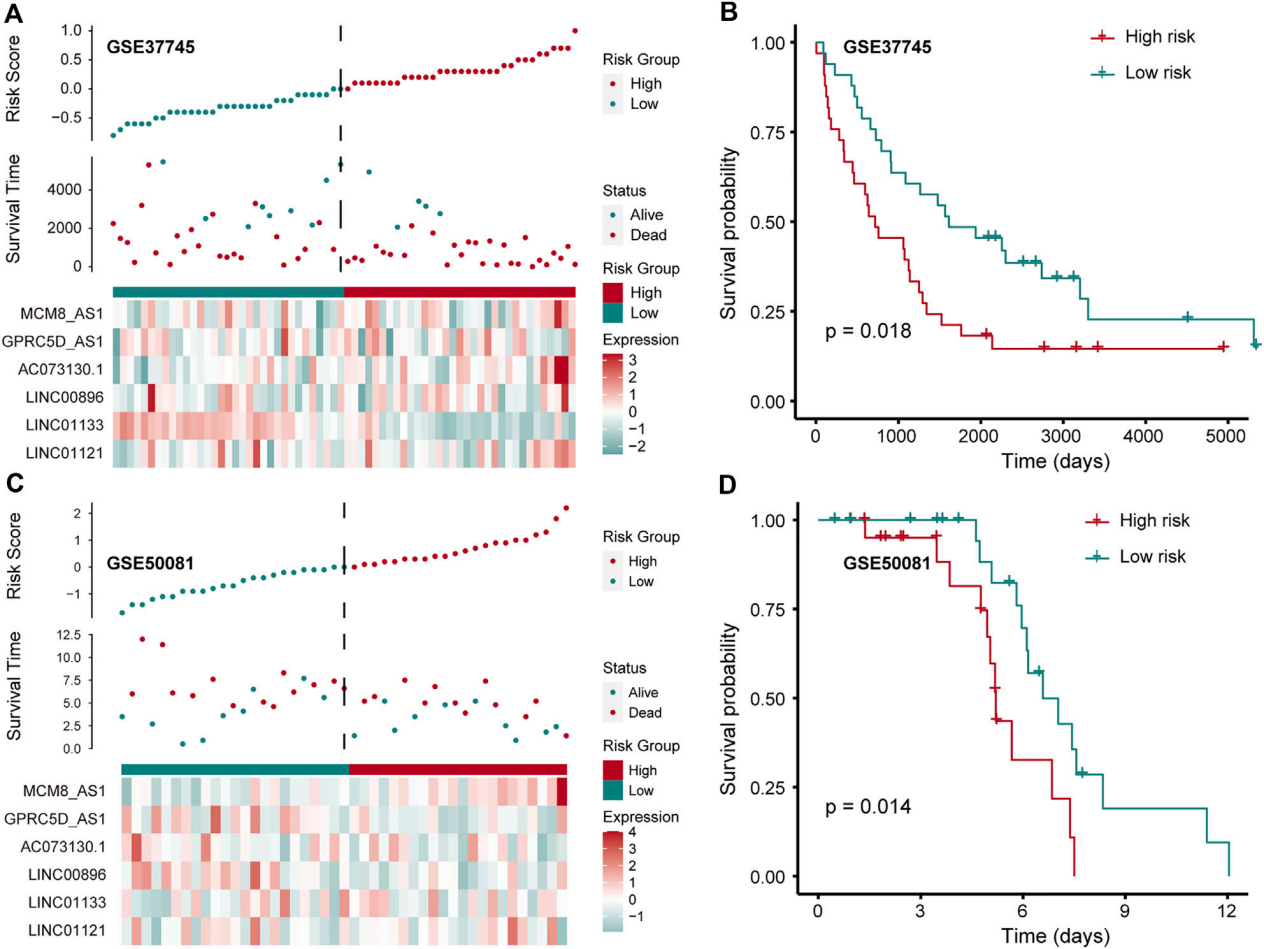
Verification of prognostic markers in public data. **(A)** The expression pattern of lncRNA and survival status and risk scores of LUSC patients for GSE37745 series. **(B)** Kaplan–Meier curve of two groups (high-/low-risk score) for GSE37745 series. Log-rank test is used to calculate statistical significance. The unit of time is days. **(C)** The same as in **(A)** but for GSE50081 series. **(D)** Kaplan–Meier curve of two groups (high-/low-risk score) for GSE50081 series. The unit of time is days.

To further confirm that the pr-lncRNA signature is a robust biomarker in LUSC, we collected the expression profiles and clinical information of two sets (GSE37745 and GSE50081) including LUSC samples from public databases for prognostic marker testing. Furthermore, we divided samples from GSE37745 series into high-risk group (*n* = 33 LUSC samples) and low-risk group (*n* = 33 LUSC samples) based on median risk score. We found that only six pr-lncRNAs were detected in the microarray matrix, and the risk score distribution, survival status, and lncRNA expression of all patients were consistent with those observed in TCGA cohort (Figure 4A). There is a significant difference in the OS between the two groups, and the risk score was also identified as a poor prognosis marker (*p* = 0.018) (Figure 4B). In the set of GSE50081 series, 45 patients of LUSC were divided into high-risk group (*n* = 22) and low-risk group (*n* = 23). The results of log-rank test also showed that the risk score was significantly correlated with OS (Figure 4C,D). All these further supported that the pr-lncRNA signature was a robust prognosis indicator in LUSC.

### Immune Cell Components Regulated by Pr-LncRNAs Support Tumor Progression

**FIGURE 5:**
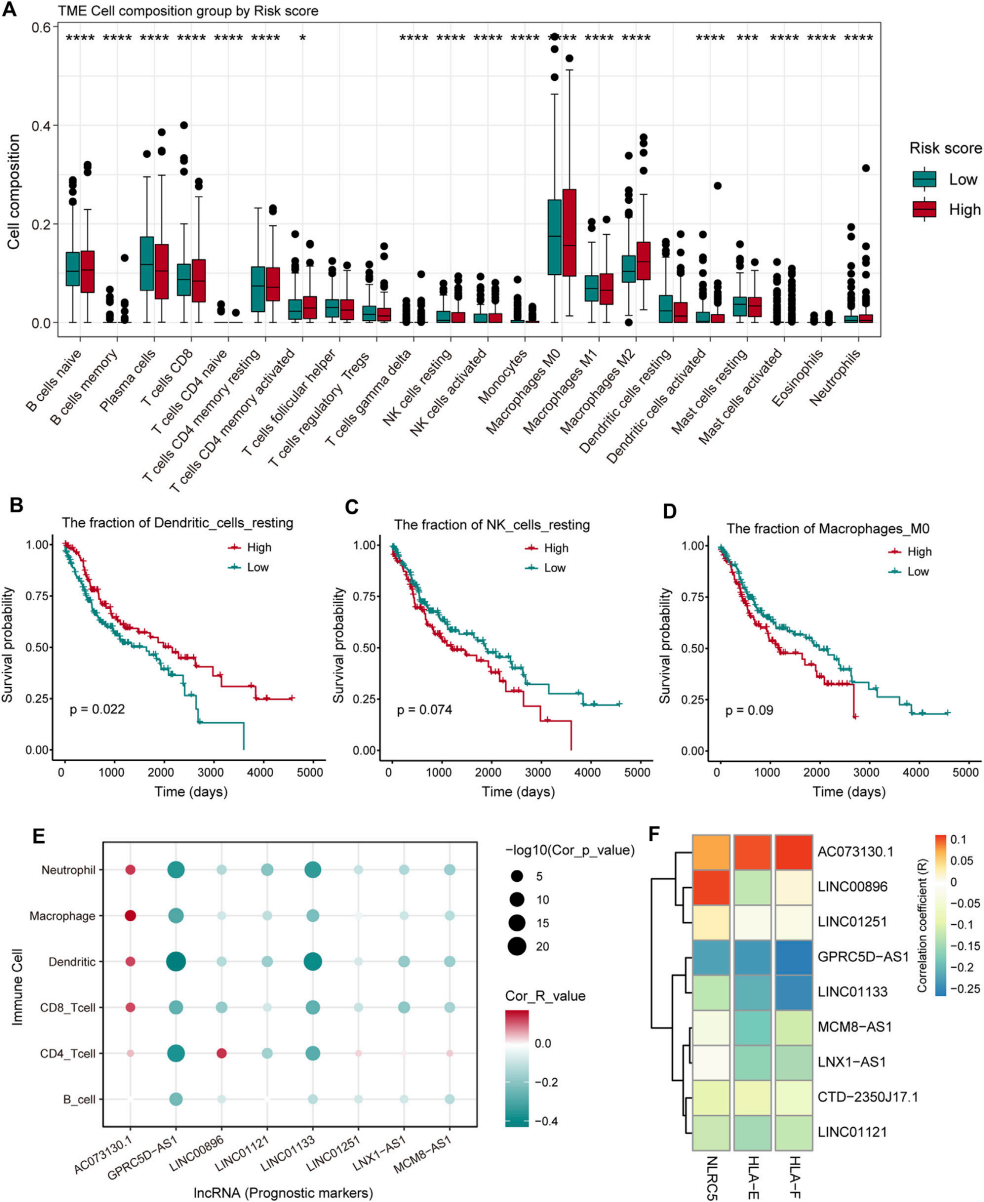
The analysis of the relationship between pr-lncRNAs and tumor immune activity. **(A)** The infiltration abundance of 22 types of leukocytes between the high- and low-risk groups is displayed by a boxplot. The *p*-values were calculated by the Mann–Whitney *U* test. **(B–D)** Kaplan–Meier curve of the two groups (high-/low-infiltration abundance) for resting dendritic cells, resting natural killer (NK) cells, and macrophage M0. Log-rank test is used to calculate statistical significance. **(E)** The bubble chart shows the correlation between the infiltration abundance of the six immune cells and the expression of pr-lncRNA. **(F)** The relationship between the expression of genes encoding major histocompatibility complex I (MHC I) molecules and that of pr-lncRNAs is displayed by a heat map. The stronger the correlation, the darker the color.

To investigate the effect of the nine pr-lncRNAs in the tumor immune microenvironment, we first identified the abundance of immune cell infiltration for TCGA cohort using the CIBERSORTx tool. The LUSC samples of TCGA cohort were divided into two groups based on the risk score of each sample, and the statistical difference in the infiltration abundance of 22 types of leukocytes between the two groups was calculated. We found that the infiltration abundance of multiple immune cells is significantly different in the high- and low-risk groups (Figure 5A). For example, the infiltration abundance of memory CD4^+^ T cells was up-regulated in the high-risk group, the infiltration abundance of macrophage M0 was up-regulated in the high-risk group, and the infiltration abundance of resting dendritic cells was down-regulated in the high-risk group. By combining with the patient’s survival information, we found that the infiltration abundance of the three cell types [resting dendritic cells, resting natural killer (NK) cells, and macrophage M0] were significantly related to the patient’s prognosis (Figure 5B–D). High infiltration abundance of resting dendritic cells was associated with better patient prognosis (Figure 5B), which is consistent with the phenomenon of low expression of resting dendritic cells in high-risk samples. We found that the expression abundance of resting NK cells can be used as a poor prognostic marker (Figure 5C), which may be due to the low solubility of resting NK cells to target cells (Bryceson et al., 2006). Moreover, the high fraction of macrophage M0 was significantly associated with poor patient prognosis (Figure 5D), which is consistent with a previous study suggesting that macrophage polarization plays a key role in promoting tumor progression (Mantovani et al., 2002). These suggest that the infiltration of these immune cell types could support tumor progression. Furthermore, we systemically analyzed the correlation between pr-lncRNA expression and immune cell infiltration abundance. For the pr-lncRNAs, *AC073130.1*, *GPRC5D-AS1*, and *LINC01133* exhibited significant associations with multiple immune cells, i.e., CD4^+^ T cell, CD8^+^ T cell, dendritic cell, macrophage, and neutrophil, suggesting that the three pr-lncRNAs may be immune-related lncRNAs for LUSC (Figure 5E). We also found that the pr-lncRNA *GPRC5D-AS1* showed negative correlations with the expression levels of major histocompatibility complex I (MHC I) and MHC II (Figure 5F and Supplementary Figure S2A) that assist in tumor cell recognition and antigen presentation (Lauss et al., 2017), indicating that a high expression of *GPRC5D-AS1* may reduce tumor immunogenicity.

### The Cascade of Pr-LncRNAs Regulates the Carcinogenic Mechanisms for LUSC

**FIGURE 6:**
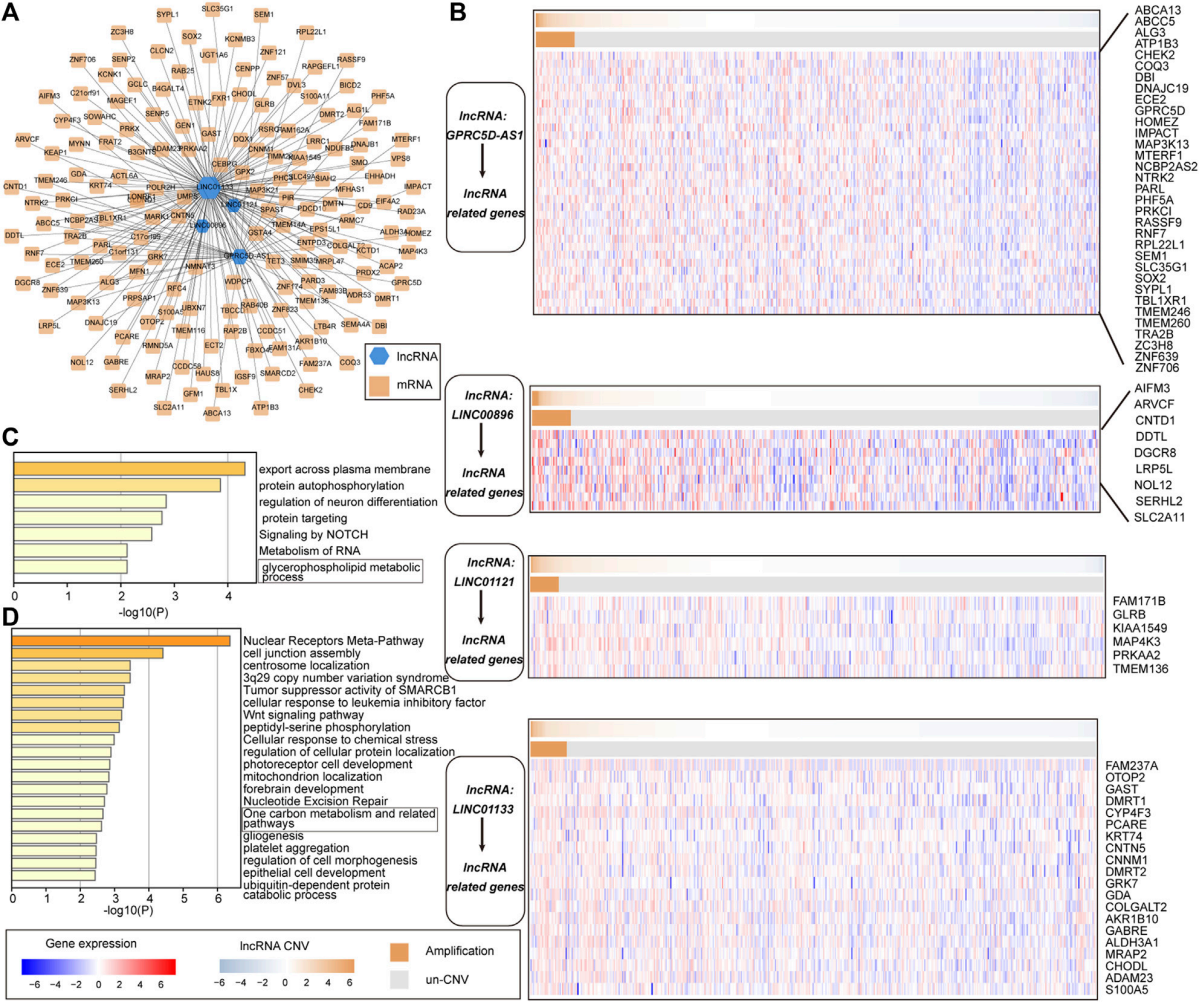
The cascade response of pr-lncRNA-CNV-mRNA carcinogenic mechanism. **(A)** pr-lncRNA-mRNA regulatory network showing the dysregulation of functional genes driven by lncRNA-CNV. The green hexagon represents lncRNA, and the yellow square represents mRNA. **(B)** Four (lncRNA-CNV) gene cascade responses. The heat map combination includes the copy number and the expression of lncRNA-related genes for the four pr-lncRNAs. The first row of each combination represents the copy number amplitude of lncRNA, and the second row represents the copy number amplification and deletion of lncRNA. **(C–D)** The enrichment results for genes related to lncRNA GPRC5D-AS1 and LINC01133 are displayed by bar graphs, colored by *p*-values.

To better understand the biological functions and regulatory mechanisms of pr-lncRNA-CNV, we explored the pr-lncRNA-mRNA regulatory mode. In determining the regulatory relationship of lncRNA-mRNA, we required the co-expression of lncRNA and mRNA, and mRNA was expressed significantly different between the CNV group and the control group as determined by the lncRNA. We identified 183 lncRNA-mRNA regulatory axes containing four pr-lncRNAs (*GPRC5D-AS1*, *LINC00896*, *LINC01121*, and *LINC01133*) and 167 mRNAs to construct the pr-lncRNA-mRNA network (Figure 6A). Furthermore, a series of lncRNA-CNV gene cascade reactions were proposed, and the physiological functions of its regulation were analyzed. The lncRNA *GPRC5D-AS1* with CNVs driving the dysregulation of downstream cancer genes such as *PRKCI*, *SEM1*, *TBL1XR1*, *IMPACT*, and *RPL22L1* further disturbed the activities of NOTCH signaling pathway that is usually inactivated in LUSC (Katoh and Katoh, 2020) (Figure 6B,C). Similarly, the lncRNA *LINC01133* may regulate the activity of tumor suppressor pathways by dysregulating *ACTL6A*, *SMARCD2*, *SMO*, *PHC3*, and *CENPP* (Figure 6B,D). It is worth noting that *LINC01133* has been shown to be closely related to the invasion and malignant proliferation of human non-small cell lung cancer (Zhang et al., 2020; Geng et al., 2021). The lncRNA *LINC00896* is associated with copy number variation syndrome by regulating the corresponding target gene (Supplementary Figure S2B). Taken together, all these results provide novel insights for understanding the function of lncRNA-CNV and the pathogenesis based on these lncRNA-CNV.

## Discussion

In this study, we integrated multiple omics data to identify the prognostic-related lncRNAs driven by CNV and define the cascade response of lncRNA-CNV-mRNA carcinogenic functions. Based on the principle of CNV dysregulated gene expression, we have identified 372 lncRNAs whose expression levels vary with copy number amplitude. Through the combined Cox regression algorithm and LASSO algorithm, nine CNV-driven lncRNAs were identified as prognostic markers. Immune cell infiltration analysis revealed the composition of the immune microenvironment for LUSC and the leukocytes associated with the patient’s survival risk. Moreover, the correlation between the nine pr-lncRNAs and immune cell types has been revealed, emphasizing the important role of *AC073130.1*, *GPRC5D-AS1*, and *LINC01133* in immune disorders. We next identified mRNAs driven by lncRNA-CNV and defined the function of lncRNA with CNV, which highlight the novel mechanism of non-coding RNA with CNV driving oncogenic function in LUSC.

In the past decades, with the development of high-throughput sequencing dataset and technologies (Yu et al., 2018; Yu et al., 2021), the integrated analysis of multiple omics data to reveal the pathogenesis of cancer has become the norm. For example, Zheng et al. (2020) identified three lncRNA prognostic characteristics of ovarian cancer based on genome-wide CNV. Although this study is also based on genome-wide CNV to identify prognostic-related lncRNA, it did not reveal the functions and regulatory mechanisms of pr-lncRNA. The analysis of pr-lncRNA’s involvement in tumor immune infiltration and carcinogenic function cascade is an important feature of this study.

The lncRNAs *GPRC5D-AS1* and *LINC01133* were emphasized in this study as prognostic markers that play an important role in immune cell infiltration and carcinogenic mechanisms. The expression levels of *GPRC5D-AS1* and *LINC01133* were negatively correlated with the infiltration of multiple immune cells (CD4^+^/CD8^+^ T cell, dendritic cell, and neutrophil), suggesting that a high expression of *GPRC5D-AS1* and *LINC01133* can inhibit cell infiltration. This provides novel therapeutic target for the development of immune checkpoint therapy.

In summary, we provided prognostic-related lncRNA driven by CNV for LUSC and revealed the regulatory mechanism of lncRNA-CNV-mRNA oncogenic function. By revealing the function of pr-lncRNA, potential targets that can be used for immunotherapy have been identified. Taken together, our research provides useful theoretical guidance for the clinical diagnosis and treatment for LUSC.

## Data Availability

The original contributions presented in the study are included in the article/Supplementary Material; further inquiries can be directed to the corresponding author.
