# Supplementary material for: Characterizing the Copy Number Variation of Non-Coding RNAs Reveals Potential Therapeutic Targets and Prognostic Markers of LUSC
Source: Front Genet. 2021 Dec 1;12:779155. doi: 10.3389/fgene.2021.779155 (PMC8672037; doi:10.3389/fgene.2021.779155)
Supplement: Supplementary file 1 [file DataSheet1.docx]

**Supplementary Figures**

**
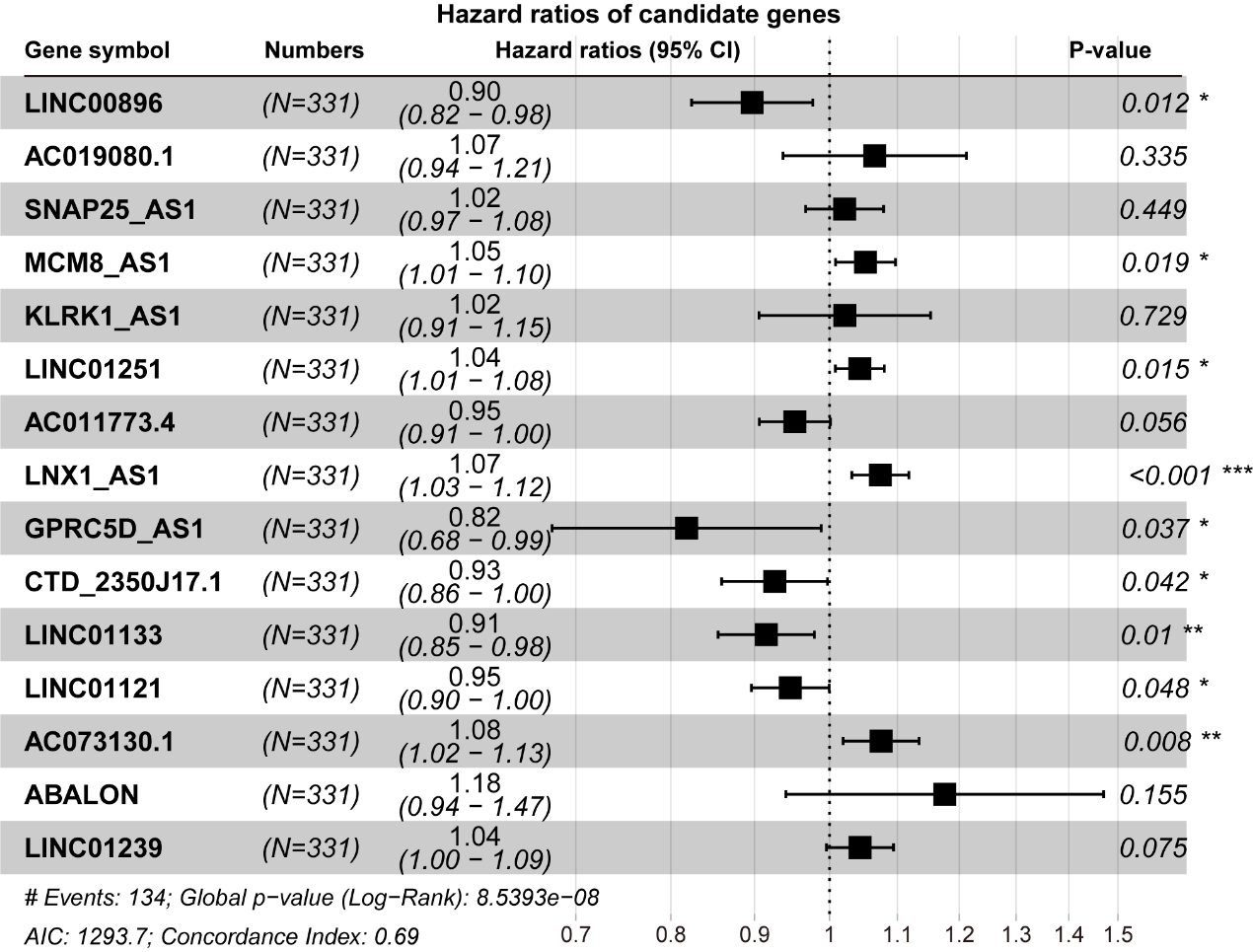
**

**Figure S1.** Forest plots for multivariate Cox regression models.

**
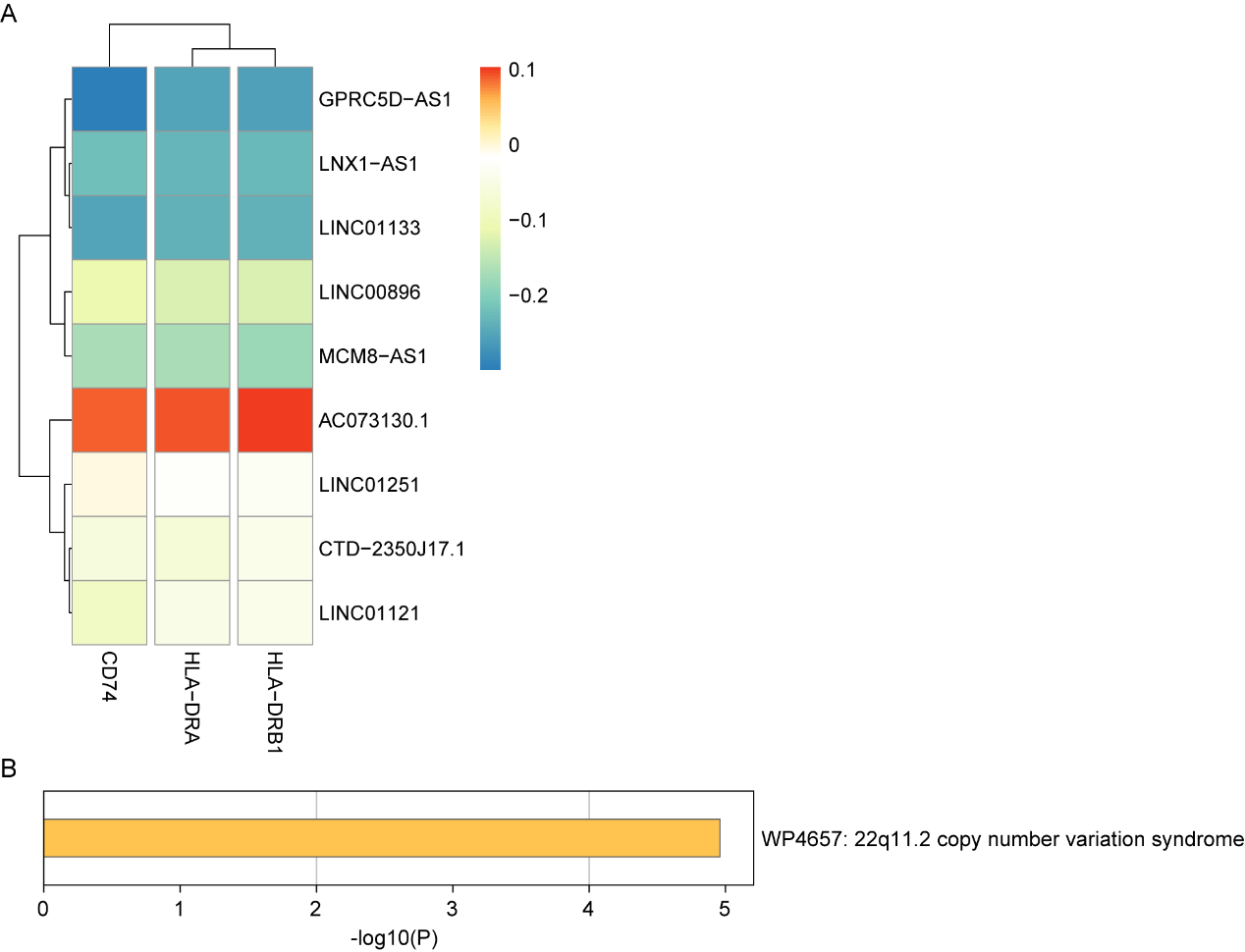
**

**Figure S2.** (A) The relationship between the expression of genes encoding MHC II molecules and that of pr-lncRNAs is displayed by heat map. The stronger the correlation, the darker the color. (B) The enrichment results for genes related to lncRNA LINC00896 are displayed by bar graphs, colored by p-values.

**Supplementary Tables**

**Table S1:** **Sample information of GSE37745 and GSE50081 series**

| GEO Accession number | Platform | Tissue | LUSC sample size | Other subtypes sample size |
| --- | --- | --- | --- | --- |
| GSE37745 | GPL570 | Lung | 66 | 130 |
| GSE50081 | GPL570 | Lung | 45 | 136 |
